# Serological and Molecular Characterization of Avian Metapneumovirus in Chickens in Northern Vietnam

**DOI:** 10.3390/vetsci8100206

**Published:** 2021-09-24

**Authors:** Van-Giap Nguyen, Hee-Chun Chung, Hai-Quynh Do, Thanh-Trung Nguyen, Thi-Bich-Phuong Cao, Ha-Thai Truong, Thi-Ngan Mai, Thi-Trinh Le, Thi-Hoa Nguyen, Thi-Luyen Le, Thi-My-Le Huynh

**Affiliations:** 1 Department of Veterinary Microbiology and Infectious Diseases, Faculty of Veterinary Medicine, Vietnam National University of Agriculture, Hanoi 100000, Vietnam; nvgiap@vnua.edu.vn (V.-G.N.); ctbphuong@vnua.edu.vn (T.-B.-P.C.); ththai@vnua.edu.vn (H.-T.T.); mtngan@vnua.edu.vn (T.-N.M.); 2 Department of Veterinary Medicine Virology Lab., College of Veterinary Medicine and Research Institute for Veterinary Science, Seoul National University, Seoul 08826, Korea; heeskyi@snu.ac.kr (H.-C.C.); quynhdohai@gmail.com (H.-Q.D.); 3 Institute of Genome Research, Vietnam Academy of Science and Technology, Hanoi 100000, Vietnam; 4 Department of Pharmacology, Toxicology, Internal Medicine and Diagnostics, Faculty of Veterinary Medicine, Vietnam National University of Agriculture, Hanoi 100000, Vietnam; nguyenthanhtrung@vnua.edu.vn; 5 Vietnam Green Vet Joint Stock Company, Hanoi 100000, Vietnam; trinhle24061996@gmail.com; 6 Key Laboratory for Veterinary Biotechnology, Vietnam National University of Agriculture, Hanoi 100000, Vietnam; hoanguyen2405@gmail.com (T.-H.N.); leluyentyc@gmail.com (T.-L.L.)

**Keywords:** avian Metapneumovirus, serology, molecular characterization, chickens, Vietnam

## Abstract

Avian Metapneumovirus (aMPV) is a causative agent of respiratory disease complex in turkeys and chickens that has recently been detected in Vietnam. Due to its novelty, this study was conducted to elucidate the distribution of aMPV in several provinces in northern Vietnam. By the application of Enzyme-Linked Immunosorbent Assay (ELISA) and nested Reverse Transcription-Polymerase Chain Reaction (RT-PCR), this study demonstrated the circulation of aMPV in 12 out of 14 cities/provinces with positive rates of 37.6% and 17.2%, respectively. All nested RT-PCR positive samples were aMPV subgroup B. By pairing the detection results with age groups, it was observed that aMPV infections occurred in chickens of all ages. Additionally, by genetic characterization, aMPV strains were demonstrated to not be attenuated vaccine viruses and to belong to at least two genetic clades. Overall, the obtained results provided insights into the prevalence of aMPV and indicated a greater complexity of respiratory diseases in chickens in Vietnam.

## 1. Introduction

During the transformation from small-scale livestock production to high-density industrial farming, common respiratory diseases among poultry have been occurring regularly. Poultry may become infected with several types of respiratory illnesses caused by bacteria, viruses, and mycoplasma [1,2]. In terms of viral diseases, illnesses caused by avian Metapneumovirus (aMPV) are believed to be common in all chicken-producing countries and are responsible for huge economic losses of millions of USD per year in poultry production [3].

aMPV is a member of the genus *Metapneumovirus* that can infect both domesticated (e.g., turkeys, chickens, and ducks) [4,5] and wild birds [6,7], among which turkeys and chickens are the two most susceptible species. In further detail, the virus attacks the mucosa of the upper respiratory tract [8,9], causing nasal exudates, frothy eye, conjunctivitis, and swelling of the head–facial sinuses [10]. The disease has a low mortality rate of 2–5% that can increase to 25% in the case of co-infection [11,12]. In addition to causing respiratory disease, aMPV also affects reproductive functions such as reduced egg production and eggshell quality [12,13,14] and can cause abnormalities in the testes, thus reducing the quality of semen in roosters [15].

Regarding the spatial distribution, since first reported in the 1980s, aMPV has been present in Africa [16], the US [17], Europe [18], and Asia [19]. There is no available information on diseases caused by aMPV in Southeast Asia other than in Malaysia [20]. In Vietnam, aside from respiratory pathogens such as MG (*Mycoplasma gallisepticum*), ORT (*Ornithobacterium rhinotracheale*), and IBV (Infectious bronchitis virus) [21,22,23], aMPV has just recently been detected in chickens with respiratory symptoms [24]. Due to its novelty, many aspects of its epidemiology remain poorly understood. As a result, this study attempted to investigate the sero- and viral-positivity rates of aMPV and evaluate the role of aMPV infection in respiratory disease complexes of chickens raised in northern Vietnam.

## 2. Materials and Methods

### 2.1. Farm Selection, Sample Collection, and Processing

For this study, commercial layer farms and indigenous slow-growing broilers farms in which the aMPV vaccine had not been employed were selected. Furthermore, to enhance the likelihood of detecting aMPV, farms were narrowed down to those with respiratory problems. Due to the relatively short viral shedding time [25,26], respiratory swabs or respiratory organs were collected from chickens showing mild to severe clinical signs from each farm. For blood collection, chickens less than three weeks old were excluded owing to the potential for maternal-derived aMPV-specific antibodies [27].

Choanal cleft swab was taken from each bird, then put into Eppendorf tubes containing 0.5 mL of phosphate-buffered saline. At necropsy, organs of the respiratory system including nasal turbinate, larynx, trachea, lungs, and air sacs were collected from each bird. The pooled organs were minced, manually homogenized to a 10% suspension in phosphate-buffered saline by grinding with a mortar and pestle. The suspension was centrifuged at 4000 rpm for 5 min to collect the supernatant. For blood collection, a 3-mL syringe was used to take 1 mL of blood from the brachial wing vein, placed on a slanted surface for 10 min to allow for clotting. After centrifugation at 4000 rpm for 5 min, serum samples were obtained. All samples (serum, tissue supernatant) were stored at −30 °C.

A total of 138 farms were sampled from 14 northern provinces and cities. From these, 256 tissues/swabs and 327 serum samples were tested for aMPV nucleic acid and for antibodies against aMPV, respectively. In some farms, only one type of sample (either tissue/swab or serum) was available; thus, out of the 138 farms, only 34 had samples tested by both nested RT-PCR and ELISA methods, while 15 farms were tested solely by ELISA and 89 farms solely by nested RT-PCR assay.

### 2.2. Serology

Specific antibodies against aMPV were detected by indirect ELISA with the IDVET_MPVS-5P kit (IDvet, France) following the manufacturer’s instructions. The ELISA plates were pre-coated with purified aMPV antigen and were suitable for screening antibodies against aMPV subgroups A and B. The final serum dilution was 1/500 and samples were seropositive if S/P > 0.2.

### 2.3. Detection of aMPV by RT-Nested PCR

The total RNA and DNA from samples were extracted with the Patho Gene-spin DNA/RNA Extraction kit (17154, iNtRON Biotechnology, Gyeonggi, Korea), following the manufacturer’s instructions. Reverse transcription was performed using the HiSenScript RH(-) RT PreMix kit (25087, iNtRON Biotechnology, Gyeonggi, Korea) at 45 °C for 60 min. The nucleic acid of aMPV was detected by nested RT-PCR following a previous publication’s methodology [28], using Maxime PCR PreMix kit, i-StarMAX II (25281, iNtRON Biotechnology, Gyeonggi, Korea). The PCR reaction was composed of 1 µL cDNA or outer PCR product, 1.0 µL aliquot of each reverse/forward primer (10 pmol/µL each), and distilled water up to 20 µL. Appendix A Table A1 shows the sequences of primers and Table A2 shows the corresponding thermal profile. The PCR products were analyzed through agarose gel electrophoresis containing 1X RedSafe Nucleic Acid Staining Solution (21141, iNtRON Biotechnology, Gyeonggi, Korea).

### 2.4. Sequencing of the Attachment Glycoprotein Gene (G Gene) and Genetic Analyses

The samples with the strongest aMPV PCR band density were selected for genetic characterization. The same PCR premix was used to amplify G gene in the format of nested PCR by combining the primer pairs shown in Appendix A Table A3. The final 20-µL PCR reaction was composed of 0.5 pmol/µL of each primer and 1 µL cDNA or first PCR product. Briefly, the first-round PCR was performed with primer pairs Gstart+/GB1- [29]. The second PCR was performed with two primer pairs: Gstart+/428.R.s2.G and 428.F.s2.G/GB1-. The thermal cycle was done using a SureCycler 8800 (Agilent) where the heating rate was “standard temperature ramp rate”, the annealing temperature was 53 °C, and the extension temperature was 68 °C. The details are shown in Appendix A Table A4. Subsequently, PCR products were sequenced by Sanger’s method (first BASE, Malaysia). The GenBank accession numbers of sequences generated in this study are shown in Table 1.

Analysis of genetic variation along the G gene was performed using SimPlot software [30] with the default options. The phylogenetic relationships between aMPVs based on the G gene sequences were reconstructed by IQTREE software version 2.1.3 [31]. The data best fit model of nucleotide substitutions was chosen automatically by specifying the command “-m TEST”. The calculation of bootstrap values was by transfer bootstrap expectation [32] with 1000 bootstrap replications.

## 3. Results

### 3.1. Seropositivity and Virus Positivity Rates of aMPV in the Northern Provinces of Vietnam

**Figure 1:**
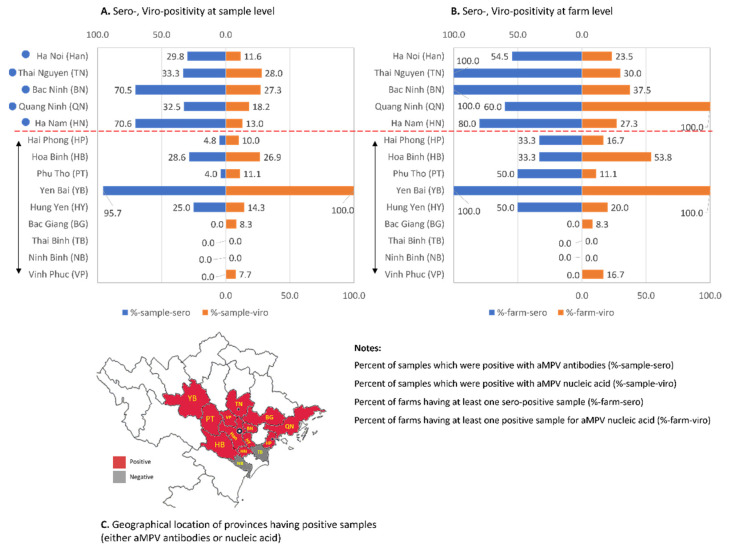
Summary of serology and virology tests for aMPV specific antibodies and nucleic acid. The detection result at the sample level (**A**) and farm level (**B**). The attached map (**C**) shows the geographical locations of the provinces that have positive samples. The double-headed arrow indicates the provinces that have fewer than five investigated farms.

This study assessed the presence of aMPV in chickens by both serological and virological tests. Of the serology results, 123/327 (37.6%) sera were positive for anti-aMPV antibodies (Figure 1A). At the farm level, the average seropositive rates were 61.2%, showing that 30/49 farms had at least one positive sample for anti-aMPV antibodies. Among localities, the sample-positivity rate was 0.0–95.7% and the farm-positivity rate was 0.0–100%. Of the provinces that had more than five investigated farms (filled circles, Figure 1), the positive sample rates were in the range 29.8–70.6% with >50–100% of farms being positive. By contrast, four provinces (Bac Giang, Ninh Binh, Thai Binh, and Vinh Phuc) had no serologically positive samples.

Aside from the serology test, clinical specimens were examined for the presence of aMPV. To improve the sensitivity, the nested RT-PCR with primers specific to the subgroups A and B [28] was applied. Forty-four of the 256 samples (17.2%) were positive for aMPV subgroup B while none were positive for subgroup A. The presence of aMPV was detected in 26.8% (33/123) farms. Of the provinces that had more than five investigated farms (filled circles, Figure 1), the viral positive rates were 11.6–28.0% (at sample level) and 23.5–100% (at the farm level). Two provinces (Thai Binh and Ninh Binh) had no positive samples, which again might largely be due to the small sample sizes. The serological and molecular diagnosis results showed that even though the detection rates of aMPV infections were low (<50% positive for both assays), the virus had been distributed widely in chickens in northern Vietnam (Figure 1C), which account for approximately 46.6% of the chicken population of the whole country (statistical data for the year 2020 on Agriculture, Forestry and Fishing, General Statistics Office of Vietnam).

### 3.2. Seropositivity and Virus Positivity Rates of aMPV According to Age-Group

**Figure 2:**
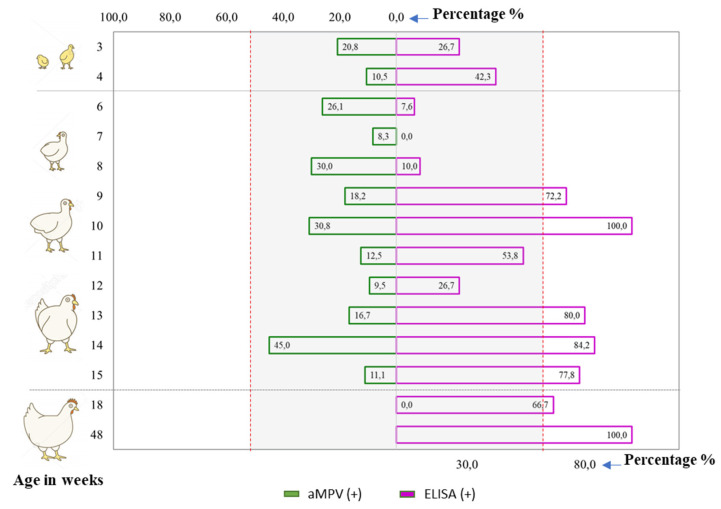
The comparison of serological and viral positivity rates by age group. Age groups that had fewer than five test samples were excluded from the comparison.

In the literature, many factors are known to affect the prevalence of aMPV such as season, breed, and age [16,33,34]. Based on the serological and molecular diagnosis results, an attempt was made to investigate the pattern of aMPV infections according to age group (shown in Figure 2).

For the serology positive rates, it was observed that almost all examined age groups were positive. Additionally, it seemed that the older group (≥9 weeks old) tended to have more positive samples (>50%) than the younger group (3–8 weeks old; <50%). For the virology positive rates, Figure 2 revealed that aMPV was detected in chickens from 3–15 weeks of age. No positive samples were detected among chickens at the production stage (≥18 weeks), but this should be interpreted with caution as it was likely due to the insufficient number of samples (Appendix A Table A5). With the current data, no trends for viro-positive rate or age group were observed. For all samples, Figure 2 reveals that it is possible to detect antibodies against aMPV and the virus in chickens from all production stages; this implies that aMPV infections occurred in chickens of all ages in northern Vietnam.

### 3.3. Genetic Characterization of the Circulating Strains of aMPV

For the genetic characterization, 14 aMPV positive samples (showing strongest PCR band density) were selected to sequence the gene encoding attachment glycoprotein (G gene). Since attenuated vaccines against aMPV have been registered in Vietnam over the past 15 years and are still available in the market, the genetic characterization of aMPV strains in this study was first inferred based on the comparison with two attenuated vaccine virus strains, namely Nemovac (Merial Animal Health) and Hipraviar (Hipra). The results shown in Table 1 reveal the differences between Vietnamese aMPV strains with the vaccine strain in 106/1230 nucleotide positions with Nemovac and 98/1071 positions with Hipraviar. Of the five strains that have sequences for only 300 nucleotides, four strains (GT1906, GK9147, GA2007, and GA1905) contained two differences compared to vaccine strains A333T and T366C; one strain (GE20117) differed from the vaccine virus at four positions, namely A91G, G201A, G254A, and A333T. Of the nine strains that have a nearly fully G gene, seven strains (Ck.1–Ck.4 and Ck.6–Ck.8) differed from Nemovac/Hipraviar at 18 positions. The other two strains (Ck.5 and Ck.9) had 90 mutations compared to the vaccine strains.

**Table 1:** Summarizing the differences in nucleotide sequences between 14 Vietnamese aMPVs and vaccine strains.

**Nucleotide position**	**20**	**22**	**57**	**91**	**102**	**138**	**142**	**176**	**200**	**201**	**214**	**241**	**251**	**254**	**306**	**314**	**333**	**356**	**366**	**430**	**443**	**449**	**451**	**474**	**485**	**500**	**505**
Nemovac_MZ574139	T	A	G	A	A	T	T	G	C	G	T	A	G	G	G	G	A	G	T	G	C	A	G	T	G	A	C
Hipraviar_MZ574138	T	A	G	A	A	T	T	G	C	G	T	A	G	G	G	G	A	G	T	G	C	A	G	T	G	A	C
GT1906_MZ574140	?	?	?	A	A	T	T	G	C	G	T	A	G	G	G	G	T	G	C	?	?	?	?	?	?	?	?
GK9147_MZ574141	?	?	?	A	A	T	T	G	C	G	T	A	G	G	G	G	T	G	C	?	?	?	?	?	?	?	?
GA2007_MZ574142	?	?	?	A	A	T	T	G	C	G	T	A	G	G	G	G	T	G	C	?	?	?	?	?	?	?	?
GA1905_MZ574144	?	?	?	A	A	T	T	G	C	G	T	A	G	G	G	G	T	G	C	?	?	?	?	?	?	?	?
GE20117_MZ574143	?	?	?	G	A	T	T	G	C	A	T	A	G	A	G	G	T	G	T	?	?	?	?	?	?	?	?
Ck.1_MZ393666	T	A	G	G	A	T	T	G	C	A	T	A	G	A	G	G	T	G	T	G	C	A	G	T	G	A	C
Ck.6_MZ393677	T	A	G	G	A	T	T	G	C	A	T	A	G	A	G	G	T	G	T	G	C	A	G	T	G	A	C
Ck.2_MZ393668	T	A	G	G	A	T	T	G	C	A	T	A	G	A	G	G	T	G	T	G	C	A	G	T	G	A	C
Ck.4_MZ393673	T	A	G	G	A	T	T	G	C	A	T	A	G	A	G	G	T	G	T	G	C	A	G	T	G	A	C
Ck.3_MZ393671	T	A	G	G	A	T	T	G	C	A	T	A	G	A	G	G	T	G	T	G	C	A	G	T	G	A	C
Ck.7_MZ393679	?	?	G	G	A	T	A	G	C	A	T	A	G	A	G	G	T	G	T	G	C	A	G	T	G	A	C
Ck.8_MZ393681	?	?	G	G	A	T	T	G	C	A	T	A	G	A	G	G	T	G	T	G	C	A	G	T	G	A	C
Ck.9_MZ393682	C	G	G	G	A	T	T	G	T	G	T	A	G	A	A	G	T	A	T	A	A	A	G	T	G	A	A
Ck.5_MZ393674	C	A	A	G	G	C	T	A	T	G	C	G	A	A	G	A	T	A	T	A	C	G	A	T	A	G	A
**Nucleotide position**	**510**	**514**	**516**	**525**	**545**	**549**	**568**	**572**	**576**	**577**	**580**	**585**	**600**	**603**	**618**	**619**	**627**	**644**	**645**	**654**	**656**	**661**	**687**	**690**	**699**	**708**	**718**
Nemovac_MZ574139	A	A	G	T	C	T	C	T	T	T	A	G	T	T	G	C	C	G	C	T	T	A	T	A	C	T	A
Hipraviar_MZ574138	A	A	G	T	C	T	C	T	T	T	A	G	T	T	G	C	C	G	C	T	T	A	T	A	C	T	A
Ck.1_MZ393666	C	A	G	T	C	T	C	T	T	T	G	G	T	T	A	C	C	G	C	T	T	A	T	A	C	T	A
Ck.6_MZ393677	C	A	G	T	C	T	C	T	T	T	G	G	T	T	A	C	C	G	C	T	T	A	T	A	C	T	A
Ck.2_MZ393668	C	A	G	T	C	T	C	T	T	T	G	G	T	T	A	C	C	G	C	T	T	A	T	A	C	T	A
Ck.4_MZ393673	C	A	G	T	C	T	C	T	T	T	G	G	T	T	A	C	C	G	C	T	T	A	T	A	C	T	A
Ck.3_MZ393671	C	A	G	T	C	T	C	T	T	T	G	G	T	T	A	C	C	G	C	T	T	A	T	A	C	T	A
Ck.7_MZ393679	C	A	G	T	C	T	C	T	T	T	G	G	T	T	A	C	C	G	C	T	T	A	T	A	C	T	T
Ck.8_MZ393681	C	A	G	T	C	T	C	T	T	T	G	G	T	T	A	C	C	G	C	T	T	A	T	A	C	T	A
Ck.9_MZ393682	A	A	A	C	T	C	C	C	C	C	G	A	C	C	G	C	A	A	T	C	C	G	C	G	C	C	A
Ck.5_MZ393674	A	G	G	T	C	C	T	T	T	T	G	G	T	C	A	A	A	G	C	T	T	G	T	G	C	C	A
**Nucleotide position**	**719**	**730**	**736**	**742**	**745**	**746**	**753**	**756**	**772**	**793**	**805**	**808**	**810**	**821**	**826**	**832**	**837**	**840**	**845**	**846**	**870**	**873**	**881**	**892**	**893**	**896**	**900**
Nemovac_MZ574139	T	C	G	T	G	G	A	A	C	A	A	A	G	G	C	G	C	T	A	A	A	A	C	C	C	T	C
Hipraviar_MZ574138	T	C	G	T	G	G	A	A	C	A	A	A	G	G	C	G	C	T	A	A	A	A	C	C	C	T	C
Ck.1_MZ393666	T	C	G	T	G	G	A	A	C	A	A	A	G	G	C	G	C	T	G	A	A	A	C	C	C	T	C
Ck.6_MZ393677	T	C	G	T	G	G	A	A	C	A	A	A	G	G	C	G	A	T	G	A	A	A	C	C	A	T	C
Ck.2_MZ393668	T	C	G	T	G	G	A	A	C	A	A	A	G	G	C	G	C	T	G	A	A	A	C	C	C	T	C
Ck.4_MZ393673	T	C	G	T	G	G	A	A	C	A	A	A	G	G	C	G	C	T	G	A	A	A	C	C	C	T	C
Ck.3_MZ393671	T	C	G	T	G	G	A	A	C	A	A	A	G	G	C	G	C	T	G	A	A	A	C	C	C	T	T
Ck.7_MZ393679	T	C	G	T	G	G	A	A	C	A	A	A	G	G	C	G	C	T	G	A	A	A	C	C	C	T	C
Ck.8_MZ393681	T	C	G	T	G	G	A	A	T	A	A	A	G	G	C	G	C	T	G	A	A	A	T	T	C	T	C
Ck.9_MZ393682	A	A	A	C	A	G	A	G	C	A	G	A	G	A	C	A	C	C	A	G	A	A	C	C	C	C	C
Ck.5_MZ393674	T	A	G	T	A	A	C	G	C	G	G	G	A	A	T	G	C	C	A	A	G	G	C	C	C	T	C
**Nucleotide position**	**914**	**915**	**926**	**938**	**961**	**972**	**975**	**977**	**979**	**980**	**985**	**1003**	**1010**	**1029**	**1032**	**1038**	**1068**	**1088**	**1132**	**1137**	**1150**	**1156**	**1187**	**1201**	**1212**		
Nemovac_MZ574139	C	A	C	T	A	A	T	C	A	T	A	A	T	T	T	T	A	C	C	T	T	G	G	G	G		
Hipraviar_MZ574138	C	A	C	T	A	A	T	C	A	T	A	A	T	T	T	T	A	?	?	?	?	?	?	?	?		
Ck.1_MZ393666	C	A	C	T	A	A	T	C	A	T	A	A	T	T	T	T	A	C	C	T	T	G	G	G	G		
Ck.6_MZ393677	C	A	C	T	A	A	T	C	A	T	A	A	T	T	T	T	A	C	C	T	T	G	G	G	G		
Ck.2_MZ393668	C	A	C	T	A	A	T	C	A	T	A	A	T	T	T	T	A	C	C	T	T	G	G	G	G		
Ck.4_MZ393673	C	A	C	T	A	A	T	C	A	T	A	A	T	T	T	T	A	C	C	T	T	G	G	G	G		
Ck.3_MZ393671	C	A	C	T	A	A	T	C	A	T	A	A	C	T	T	T	A	C	C	T	T	G	G	G	G		
Ck.7_MZ393679	C	A	C	T	A	A	T	C	A	T	A	A	T	T	T	T	A	C	C	T	T	G	G	?	?		
Ck.8_MZ393681	C	G	C	T	A	A	T	C	A	T	A	A	T	T	T	T	A	C	C	T	T	G	G	?	?		
Ck.9_MZ393682	A	A	T	C	A	G	C	T	G	C	G	G	T	C	C	C	G	T	T	T	T	A	C	A	G		
Ck.5_MZ393674	C	A	T	C	G	A	C	C	A	T	A	A	T	T	T	T	A	C	T	C	C	A	G	G	A		

Note: Sequences are arranged in the order of increasing differences from Nemovac. Missing nucleotide sequences are marked “?” and the positions of nucleotide changes between the 14 strains sequenced as compared with the vaccine strain are highlighted in yellow. For easy visualization, sequences belong to different groups were color in blue, black and red.

**Figure 3:**
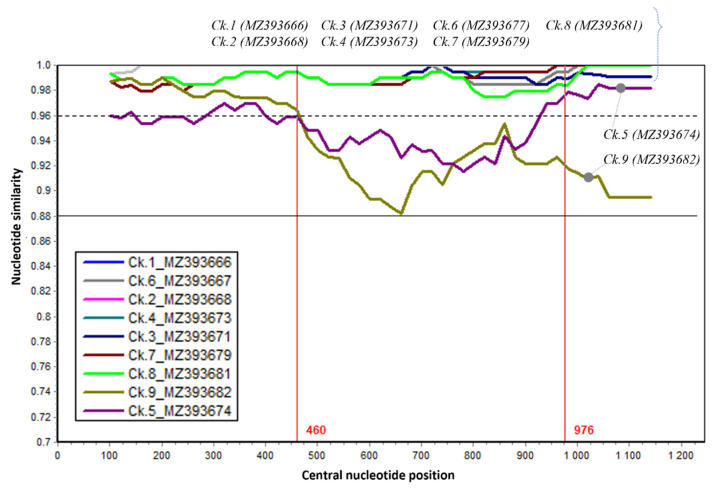
The sequence similarity of the G gene between nine Vietnamese aMPVs and the Nemovac vaccine virus. Nemovac was used as a query. Each plotted point is the percentage genetic similarity within a 200-nucleotide-wide sliding window centered on the position plotted with a step size of 20 nucleotides and Kimura 2-parameter. Some regions had similar levels of similarity with the query sequence, thus those line plots are overlapped and obscured.

The genetic comparison with a vaccine strain was further evaluated by nucleotide similarity along the G gene (Figure 3).

It was revealed that the G gene contained a region (nucleotide 1–460) that has high similarity (>96%) between strains (dashed line, Figure 3). The greatest genetic dissimilarity between strains was observed in the nucleotide region 460–976. These results once again proved the circulation of field aMPV strains among chicken farms in the northern provinces of Vietnam.

### 3.4. Phylogenetic Classification of Vietnamese aMPV Strains

**Figure 4:**
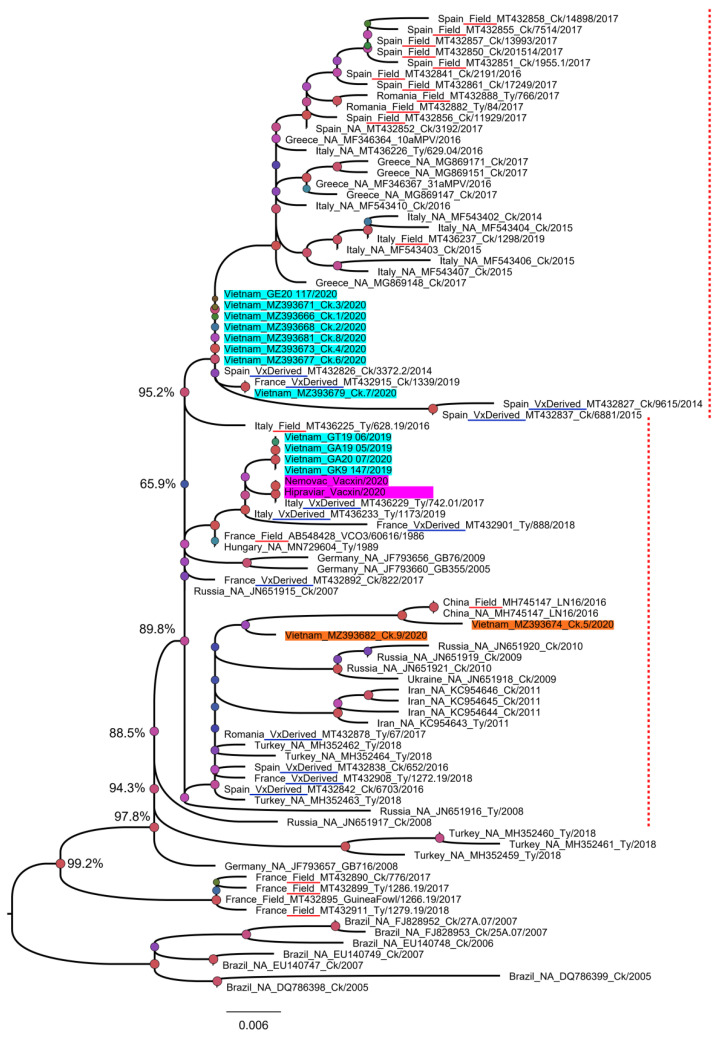
Phylogenetic tree based on a partial sequence of the G gene. The tree was reconstructed based on the sequences from nucleotides 67–366 of 89 aMPV strains (16 were generated in this study (shaded in cyan and orange for swabs/tissue samples, and pink for commercial vaccines), and 73 were retrieved from GenBank). The strains known to be field viruses or derived from the vaccine were according to a previous publication [35] and are underlined in red and blue, respectively. The labels of reference strains are given in the following format of country_field/vaccine-derived/unknown (NA)_GenBank_strain name_year of collection. The dashed red line marks the mixed branch of the field strain (Field) and the vaccine-derived strain (VxDerived). Numbers at a given node represent bootstrap confidence values; the scale bar indicates the number of nucleotide substitutions per site.

To date, the majority of aMPV sequences deposited in GenBank are partial sequences (3′ or 5′ end) of the G gene. To classify Vietnamese aMPVs to the previous known field-, vaccine-, and vaccine-derived aMPV strains [35], the phylogenetic tree was first reconstructed based on the partial G gene (nucleotides 67–366) (Figure 4). Based on the branching pattern, at least two genetic clades of an aMPV subgroup B were observed: the clade that contained only field strains (near the root of the phylogenetic tree) and the mixed clade that consisted of field strains, vaccine strains, and vaccine-derived strains (dashed red lines, Figure 4). Accordingly, 14/14 Vietnamese aMPV strains were classified as vaccine-derived strains.

**Figure 5:**
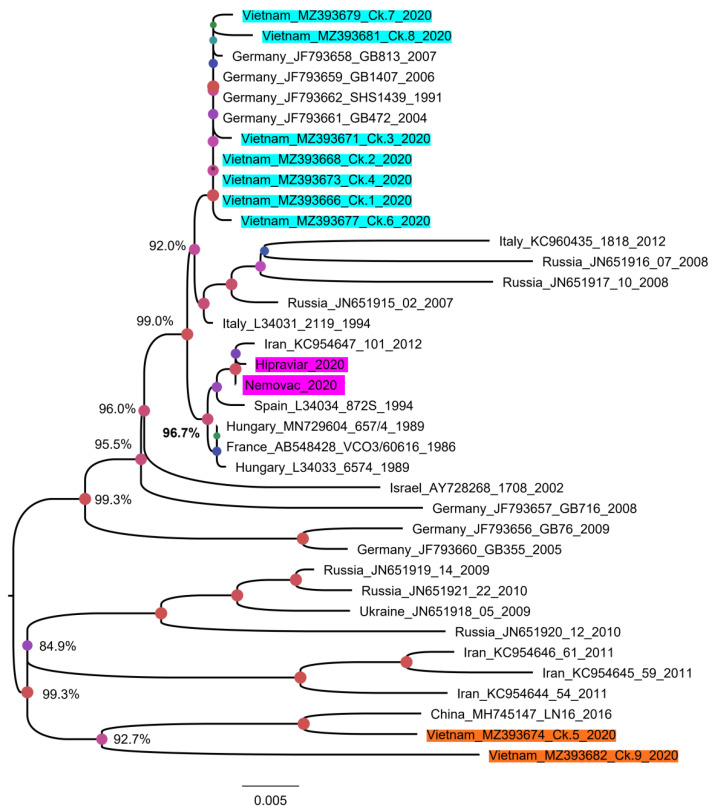
Phylogenetic tree reconstructed with a nearly complete G gene of aMPV. The tree contained 37 aMPV strains that have 1245 nucleotides of the G gene. The sequences generated in this study were shaded in cyan and orange for swabs/tissue samples, and pink for commercial vaccines. Numbers at given nodes represent bootstrap confidence values. The scale bar indicates the number of nucleotide substitutions per site.

Since the most variable genomic region of the G gene was from the 460th nucleotide, a phylogenetic tree based on the nearly complete G gene was reconstructed for a more precise classification. For this analysis, five Vietnamese aMPVs were excluded due to the partial G gene. As shown in Figure 5, nine strains of Vietnamese aMPVs are clearly separated from the branch containing the vaccine strains. In addition, based on the distant relationship between the nine Vietnamese aMPV strains on the phylogenetic tree, it was suggested that there were at least two genetic groups of aMPV circulating in northern Vietnam. Group 1 (Ck.1–Ck.4 and Ck. 6–Ck.8) was closely related to aMPVs in Europe (Germany, Italy, and Russia), while group 2 (Ck.5 and Ck.9) was related to aMPV circulating in Asia (Iran and China).

## 4. Discussion

### 4.1. The Detection Rates of aMPV Infections

To date, few studies have focused on aMPV infection in Vietnam [24]. Thus, the results from this study contribute to the understanding of the pathogens in poultry diseases in this country. Based on the serological assay, 37.6% of the chickens harbored antibodies against aMPV and the detection rates varied widely (0.0–95.7%) between the 14 investigated provinces. Although fluctuations in detection rates between geographical locations have been reported in other countries [36,37], the main reason that some northern provinces had no serologically positive samples might largely be due to the small sample sizes, which did not accurately reflect the exposure status. Of the sero-positive rates at the farm level, the average rates of 61.2% positive farms in this study was considered to be an average rate compared to other countries, such as 21.7–35.2% in Egypt [38], 30.4% in Uruguay [39], 97.3% in Korea [40], and 100% in Trinidad & Tobago [41].

In the literature, the reported prevalence of aMPV was low (<30%) in many countries; for example, it was 1.04% in Ethiopia [42], 2.8% in Greece [43], 13.3% in Jordan [16] and 25.6% in Italy [44]. The detection rates of aMPV in northern Vietnam (17.2%) demonstrated a similar low prevalence to other countries. Three main possibilities for the low aMPV detection rates were: (i) aMPV poorly replicated in the respiratory tract of chickens compared to turkeys [45,46], (ii) relatively short viral shedding time (within one week post-infection) [25,26], and (iii) low level of viral shedding (the highest was about 10^2.7^ TCID_50_/mL) [25]. Additionally, the sensitivity of the RT-PCR assay was known to affect the virus-detection results. For example, nested RT-PCR enabled the finding of aMPV in chickens 28 days post-infection [47]; meanwhile, RT-PCR could generally only detect the virus within a week post-infection [10,25,26].

Of the subgroup distribution, the aMPV subgroups A and B are generally the most common and have been detected in Asia, Europe, and South America, but not in North America; subgroup C was first discovered in North America and then France [48] and Korea [49], while subgroup D was isolated in France [50]. Simultaneous circulation of multiple subgroups is known in many countries such as Japan [19], Israel [51], Brazil [52], Nigeria [53], and China [53,54,55]. In contrast, some European countries (Italy, Romania, and Greece) have not detected subgroup A, but only subgroup B since 2017 [43,44,56]. Therefore, detecting only aMPV subgroup B circulating in northern Vietnam was not exceptional. Of the molecular based detection of aMPV, this study contained a weak point as it did not attempt to detect subgroups C and D. This was due to the lack of a high sensitive nested RT-PCR method for those subgroups, and the authors believed that subgroups C and D had limited geographical distributions [48,49,50]. Shedding more light on the presence of all four aMPV subgroups across the whole country will require expanding the study to the other ecological regions from North to South Vietnam.

### 4.2. The Detection Rates of aMPV Infections According to Age-Group

Sampling chickens of various age groups and performing serological and virological examinations enabled the finding that aMPV infections occurred in chickens of all ages in northern Vietnam. That finding was in line with several published studies that reported the detection of aMPV in chickens from different age groups, such as: 3–8 weeks [28,53], 6–16 weeks [57], or 36 weeks [58]. An explanation was that aMPV was able to infect chickens/turkeys of any age [59]. Moreover, the adaptive immune response against aMPV did not completely block infection, but limited virus replication and shedding [14,25,60,61]. The seropositivity among three-week-old chicks (Figure 2) might be due to early infections and the rapid onset of humoral immunity against aMPV at the 10th day post-infection [10,55].

### 4.3. The Genetic Characterization of Vietnamese aMPVs

The G gene was selected because it is one of the most variable genes of aMPV; it has been often selected for aMPV phylogenetic classification [62] and because the alterations at specific regions of the G protein influences the cell-mediated immune response [29]. Additionally, based on that gene, aMPV could be classified into “vaccine-derived strain” or “field strain” [35]. In brief, an aMPV strain was considered as a “vaccine-derived strain” if it fulfills two criteria of (i) having equal or less than two nucleotide differences with a reference vaccine strain, and (ii) grouped within the same phylogenetic cluster with the reference vaccine strain. The comparison with two aMPV-attenuated virus vaccines in the Vietnam market (Table 1) revealed that the nine strains (Ck.1–Ck.9) that have a nearly fully G gene had far more than two nucleotide differences with the Nemovac/Hipraviar vaccine. They belonged to distinct phylogenetic clusters (supported by high bootstrap values ≥ 99.0%) from that of the vaccine strains (Figure 5). According to the above criteria, it was safe to imply that 9/14 strains of aMPV in this study were field strains. The remaining five strains had a short sequence of G gene, and failed to separate from the previous defined vaccine-derived strains in other countries (Figure 4), thus they likely evolved from the vaccine strains.

Regarding the genetic indications of aMPV pathogenicity, the comparison between virulent aMPV strain (VCO3/60616) and attenuated virus strain (VCO3/50) identified 18 distinct nucleotides distributed in 7 of 8 structural genes and a non-protein-coding region [63]. Of these, three differences (G91A, T474T/G, and T699C) were within the G gene. As depicted in Table 1, although bearing the nucleotide sequence of the attenuated aMPV strain (474T, 699C), 9 of the 14 aMPV strains sequenced in this study had more mutations in the G gene than the attenuated vaccine strain. On the other hand, some evidence indicated that multigenic factors contribute to the virulence of aMPV [64,65] and no genetic markers for virulence were reliable due to random mutation during the in vitro attenuation process [66]. At the time when this work was submitted, an aMPV strain (Ck.51, MZ393686) was successfully isolated on chicken embryo lung and trachea mixed cultures following the previous method [28]. Genetic analyses (not shown) revealed that the Ck.51 isolate had a 97% nucleotide identity and was closely related to the Chinese LN16 isolate (MH745147) for which its pathogenicity was demonstrated in vivo [55]. Therefore, further studies are needed to elucidate the pathogenicity of aMPV strain(s) in northern Vietnam under experiment conditions.

To date, several reports [35,56] have classified aMPV subgroup B based on the partial G gene (about 171–300 nucleotides at the 3′ end) and not involving the highly diverse genomic regions of this gene as shown in Figure 3 (nucleotides 460–976). By comparing the phylogenetic tree based on the partial G gene (Figure 4) and nearly full-length G gene (Figure 5), this study suggests that the highly divergent region of the G gene must be included for genetic characterizations and classification.

## 5. Conclusions

Taken together, the present study’s results confirmed the wide distribution of aMPV subgroup B in the chicken population in northern Vietnam. The virus differed from attenuated vaccine viruses and belongs to at least two genetic clades.

## Data Availability

Data are contained within article and Appendix A.

## Appendix A

**Table A1:** List of primers used for aMPV detection.

PCR Type	Forward/Reverse	Sequences (5′-3′)	References
Outer	APV1/2.444G6-	CTGACAAATTGGTCCTGATT	[28]
	APV1.444G1+	GGGACAAGTATCYMKAT	
Inner	APV1.268.361G5-	CAAAGARCCAATAAGCCCA	[28]
	APV1.268G8+	CACTCACTGTTAGCGTCATA	
	APV1.361G9+	TAGTCCTCAAGCAAGTCCTC	

**Table A2:** Thermal profile used for aMPV detection.

PCR Type	Forward/Reverse	Thermal Profile
Outer	APV1/2.444G6-	1X (94 °C-4 min) 40X (94 °C-20 s, 50 °C-30 s, 72 °C-30 s) 1X (72 °C-5 min)
	APV1.444G1+	
Inner	APV1.268.361G5-	1X (94 °C-4 min) 40X (94 °C-20 s, 50 °C-40 s, 72 °C-60 s) 1X (72 °C-5 min)
	APV1.268G8+	
	APV1.361G9+	

**Table A3:** List of primers used for G gene sequencing.

Forward/Reverse	Sequences (5′-3′)	References
Gstart+	CAAGTATCCAGATGGGGTC	[29]
/GB1-	GTACAGCACCACTCAATCAG	
428.F.s2.G	GCTTATTGGCTCTTTGTGA	This study
/428.R.s2.G	CGGAGTTGCTGTGCTGT	

**Table A4:** Thermal profile used for G gene amplification.

PCR Type	Forward/Reverse	Thermal Profile
First PCR	Gstart+ /GB1-	1X (94 °C-4 min) 40X (94 °C-20 s, 53 °C-30 s, 68 °C-60 s) 1X (72 °C-5 min)
Second PCR-1	428.F.s2.G /GB1-	1X (94 °C-4 min) 40X (94 °C-20 s, 53 °C-30 s, 68 °C-60 s) 1X (72 °C-5 min)
Second PCR-2	Gstart+ /428.R.s2.G	1X (94 °C-4 min) 40X (94 °C-20 s, 53 °C-30 s, 68 °C-60 s) 1X (72 °C-5 min)

**Table A5:** The detection results of aMPV according to age group.

Age * (Week)	aMPV Nested RT-PCR			aMPV ELISA		
	No. Swabs/Organs	Positive	% Positive	No. of Serum	Positive	% Positive
3	24	5	20.8	15	4	26.7
4	19	2	10.5	26	11	42.3
5	3	1	33.3	9	0	0.0
6	23	6	26.1	79	6	7.6
7	12	1	8.3	5	0	0.0
8	10	3	30.0	10	1	10.0
9	11	2	18.2	18	13	72.2
10	13	4	30.8	15	15	100.0
11	16	2	12.5	26	14	53.8
12	21	2	9.5	60	16	26.7
13	6	1	16.7	5	4	80.0
14	20	9	45.0	19	16	84.2
15	9	1	11.1	9	7	77.8
16	9	0	0.0	2	2	100.0
17	1	0	0.0			
18	6	0	0.0	6	4	66.7
19	1	0	0.0			
20	3	0	0.0	2	0	0.0
21	1	0	0.0			
24	2	0	0.0			
26	1	0	0.0			
28	3	0	0.0	11	0	0.0
32	1	0	0.0			
37	1	0	0.0			
48	1	0	0.0	10	10	100.0
60	1	0	0.0			
96	1	0	0.0			
NA **	37	5	13.5			
**Total**	**256**	**44**	**17.2**	**327**	**123**	**37.6**

Notes: age groups displayed in Figure 2 were shaded. * Some age groups that had fewer than five test samples (either swabs/organs or sera) were dropped. ** NA: loss of data.
